# Molecular characterization of methicillin-resistant Panton-valentine leukocidin positive *staphylococcus aureus* clones disseminating in Tunisian hospitals and in the community

**DOI:** 10.1186/1471-2180-13-2

**Published:** 2013-01-07

**Authors:** Ben Jomàa-Jemili Mariem, Teruyo Ito, Meng Zhang, Jingxun Jin, Shanshuang Li, Boutiba-Ben Boubaker Ilhem, Hammami Adnan, Xiao Han, Keiichi Hiramatsu

**Affiliations:** 1 Laboratoire de recherche Résistance aux antimicrobiens, Faculty of Medicine of Tunis, University of Tunis El Manar, Tunis, Tunisia; 2 Department of Infection Control Science, Juntendo University, Tokyo, Japan; 3 Department of Bacteriology, Juntendo University, Tokyo, Japan; 4 Department of Microbiology, Charles Nicolle Hospital of Tunis, Tunis, Tunisia; 5 Department of Bacteriology, Habib Borguiba Hospital of Sfax, Tunis, Tunisia

## Abstract

**Background:**

The spread of MRSA strains at hospitals as well as in the community are of great concern worldwide. We characterized the MRSA clones isolated at Tunisian hospitals and in the community by comparing them to those isolated in other countries.

**Results:**

We characterized 69 MRSA strains isolated from two Tunisian university hospitals between the years 2004-2008. Twenty-two of 28 (79%) community-associated MRSA (CA-MRSA) strains and 21 of 41 (51%) healthcare-associated MRSA (HA-MRSA) strains were PVL-positive. The PVL-positive strains belonged to predicted founder group (FG) 80 in MLST and carried either type IVc SCC*mec* or nontypeable SCC*mec* that harbours the class B *mec* gene complex. In contrast, very diverse clones were identified in PVL-negative strains: three FGs (5, 15, and 22) for HA-MRSA strains and four FGs (5, 15, 45, and 80) for CA-MRSA strains; and these strains carried the SCC*mec* element of either type I, III, IVc or was nontypeable. The nucleotide sequencing of phi7401PVL lysogenized in a CA-MRSA strain JCSC7401, revealed that the phage was highly homologous to phiSA2mw, with nucleotide identities of more than 95%. Furthermore, all PVL positive strains were found to carry the same PVL phage, since these strains were positive in two PCR studies, identifying gene linkage between *lukS* and *mtp* (major tail protein) and the lysogeny region, both of which are in common with phi7401PVL and phiSa2mw.

**Conclusions:**

Our experiments suggest that FG80 *S. aureus* strains have changed to be more virulent by acquiring phi7401PVL, and to be resistant to β-lactams by acquiring SCC*mec* elements. These novel clones might have disseminated in the Tunisian community as well as at the Tunisian hospitals by taking over existing MRSA clones.

## Background

The spread of antibiotic resistance among *Staphylococcus aureus* strains is of great concern in the treatment of Staphylococcal infections. Since the first Methicillin Resistant *Staphylococcus aureus* (MRSA) strain was reported in England in 1961 [1], MRSA has become one of the most prevalent pathogens that cause nosocomial infections throughout the world. Recent reports suggest that it has become increasingly prevalent in the community as well since the 1990s [2-5]. In the 2000s, outbreaks of community-associated MRSA (CA-MRSA) strains were observed worldwide as causative agents of community-associated infections, e.g., superficial skin and soft tissue infections, urinary tract infections and pneumonia [6-9].

Methicillin resistance in MRSA is encoded by the *mecA* gene, which is carried by the SCC*mec* element, a mobile genetic element that carries methicillin resistance [10,11]. The structures of SCC*mec* elements are divergent. At least 11 types of SCC*mec* elements have been identified [12-14]. Accordingly, MRSA clones are defined by the combination of the genotype of the *S. aureus* strain and the type of SCC*mec*[15]. By using molecular epidemiological techniques, it became evident that CA-MRSA strains were distinct from those of healthcare-associated MRSA (HA-MRSA) strains. The majority of CA-MRSA strains harbour small-sized type IV or type V SCC*mec* elements and are susceptible to many antibiotics [16-18]. In contrast, HA-MRSA isolates carry one of the three types of SCC*mec* (types I, II or III) or occasionally types IV and V, and are generally multidrug resistant [6,19].

Interestingly, the majority of CA-MRSA strains that have emerged worldwide carried the *lukS*-PV and *lukF*-PV genes encoding Panton Valentine Leukocidine. Characteristic PVL-positive MRSA clones have been disseminated in each district or continent. In the United States, the ST8-SCC*mec*IVa (USA300) clone and ST1-SCC*mec*IVa (USA400) clone have been predominant. In Europe and some Asian countries, the ST80-type IVa SCC*mec* and ST59-SCC*mec*V(5C2&5) clones have been predominant, respectively. The *lukS*-PV and *lukF*-PV genes are located on bacteriophages. Since the first report of the PVL phage, the nucleotide sequences of several PVL phages have been reported [16,20-24]. Three structurally distinct PVL phages belonging to groups 1-3, have been identified to date.

We characterized the MRSA clones disseminated in Tunisian hospitals and the community. In this study, we conducted a retrospective analysis of the HA-MRSA and CA-MRSA strains isolated from two Tunisian hospitals between the years of 2004 and 2008. In order to characterize the MRSA strains, several different molecular typing methods were used: *mec*A gene PCR, SCC*mec* typing, the carriage of PVL gene and the genotyping using the *agr* locus typing, *spa*-typing and Multilocus Sequences Typing (MLST). Furthermore, the nucleotide sequence of the PVL phage carried by one strain was determined.

## Results

### Antimicrobial susceptibility

The CA-MRSA strains were resistant to gentamicin (7%), kanamycin (89%), amikacin (86%), tobramycin (18%), tetracyclines (75%), ofloxacine (11%), ciprofloxacin (36%), erythromycin (46%), clindamycin (14%) and rifampicin (4%). All strains were susceptible to pristinamycin, vancomycin, teicoplanin, trimethoprime-sulfamethoxazole and chloramphenicol. The HA-MRSA strains were resistant to gentamicin (38%), kanamycin (90%), amikacin (90%), tobramycin (26%), tetracyclines (88%), ofloxacine (30%), ciprofloxacin (45%), erythromycin (55%), trimethoprim-sulfamethoxazole (15%), chloramphenicol (7.5%), clindamycin (18%), rifampicin (32%) and fosfomycine (10%). All strains were sensitive to pristinamycin, vancomycin and teicoplanin.

### Characteristics of HA-MRSA clones

The characteristics of 41 HA-MRSA strains are summarized in Table 1. Twenty-one strains were PVL positive, while 20 strains were PVL negative. All PVL-positive strains belonged to the predicted founder group (FG, formerly called the “clonal complex”) 80 in the MLST genotype (ST80, 20 strains and ST1440, 1 strain). All strains belonged to *agr* group III, and four *spa*-types (70, 346, 435, and new) were identified among them. All PVL-positive strains carried the type IVc SCC*mec* element. In contrast, the PVL-negative clones were very diverse. Eight STs, three *agr* groups, and more than nine *spa* types were identified (Table 1). These strains carried SCC*mec* elements of type I, III, IVc, or were nontypeable (NT). Accordingly, at least eight MRSA clones (ST247-SCC*mec*I, ST1819-SCC*mec*I, ST239-SCC*mec*III, ST241-SCC*mec*III, ST5-SCC*mec*IVc, ST1-SCC*mec*NT, ST22-SCC*mec*NT and ST97-SCC*mec*NT) were identified in the 20 PVL-negative HA-MRSA strains, whereas there were only two MRSA clones, ST80-SCC*mec*IVc and ST1440-SCC*mec*IVc in PVL-positive HA-MRSA strains.

**Table 1:** Characteristics of the MRSA clones isolated from Tunisian hospitals and the community

	**ST**	**Predicted founder group (old clonal complex)**	***agr *type**	***spa *type**	**SCC *mec *type**
HA-MRSA (n=41)					
PVL-positive (n=21)	ST80(n=20)	80	III	70(n=16)	IVc(n=16)
				346(n=1)	IVc(n=1)
				435(n=2)	IVc(n=2)
				new(n=1)	IVc(n=1)
	ST1440(n=1)	80	III	70(n=1)	IVc(n=1)
PVL-negative (n=20)	ST1(n=1)	15(CC1)	III	35(n=1)	^b^NT-1(n=1)
	ST5(n=3)	5(CC5)	II	45(n=2)	IVc(n=1)
					NT-A(n=1)
				335(n=1)	IVc(n=1)
	ST22(n=1)	22	II	998(n=1)	NT-N(n=1)
	ST97(n=2)	15	I	3(n=1)	NT-B(n=1)
			I	new(n=1)	NT-B(n=1)
	ST239(n=4)	5(CC8)	I	3(n=4)	III(n=3)
	ST241(n=3)	5(CC8)	I	125(n=2)	III(n=2)
				4(n=1)	III(n=1)
	ST247(n=3)	5(CC8)	I	40(n=3)	I(n=3)
	ST1819(n=3)	5(CC8)	I	new(n=3)	I(n=3)
CA-MRSA(n=28)					
PVL-positive(n=22)	ST80(n=19)	80	III(n=19)	70(n=17)	IVc(n=15)
					NT-B(n=2)
				346(n=1)	IVc(n=1)
				new(n=1)	IVc(n=1)
	ST153(n=2)	80	III	new(n=1)	NT-B(n=1)
				70(n=1)	IVc(n=1)
	ST2563(n=1)	80	III	70(n=1)	IVc(n=1)
PVL-negative(n=6)	ST1(n=1)	15(CCI)	III	35(n=1)	NT-Bc(n=1)
	ST5(n=2)	5	II	381(n=1)	I(n=1)
				1021(n=1)	IVc(n=1)
	ST45(n=1)	45	I	^a^ND(n=1)	NT-B(n=1)
	ST80(n=2)	80	II	1021(n=1)	IVc(n=1)
			III	ND(n=1)	IVc(n=1)

^a^ND: could not be detected.

^b^NT: non typeable.

### Characteristics of CA-MRSA strains

The characteristics of the 28 isolated CA-MRSA strains are summarized in Table 1. Twenty-two strains (79%) were PVL-positive and six strains (21%) were PVL-negative. All PVL-positive strains belonged to FG80 and *agr* group III, and carried the type IVc or NT SCC*mec* element similar to the cases of PVL-positive HA-MRSA strains. Three *spa*-types (70, 346, and new) were identified among them. The PVL-negative strains belonged to four FGs (5, 15, 45, and 80), three *agr* groups, I- III, and there were more than four *spa* types (35, 381, 1021, and new). These strains carried SCC*mec* elements of type IVc or NT. As a result, five MRSA clones (ST1-SCC*mec*NT, ST5-SCC*mec*I, ST5-SCC*mec*IVc, ST45-SCC*mec*NT and ST80-SCC*mec*IVc) were identified in six PVL-negative CA-MRSA strains.

### SCC*mec* elements identified in Tunisian MRSA

As listed in Table 1, the SCC*mec* type of 59 out of 69 MRSA strains were classified by one of the extant types. All PVL-positive HA-MRSA strains and the majority of CA-MRSA strains carried type IV SCC*mec* of subtype c. Three PVL-positive CA-MRSA strains carried class B *mec,* but no *ccr* genes were identified so far. We expressed this as “NT-B”. SCC*mec*NT-B was identified in three PVL-negative strains belonging to ST45 and ST97. The SCC*mec* elements of the other four strains were expressed as follows: NT-1 (type 1 *ccr* positive, the class of the *mec* gene complex could not be determined), NT-A (*ccr* genes were not identified, but it carried the class A *mec* gene complex), NT-N (neither the *ccr* genes nor the *mec* gene complex could be identified), and NT-Bc (two *ccr* genes, types 1 and 2, were identified, and the class B *mec* gene complex was identified).

### The characteristics of lysogenized PVL phage

**Figure 1:**
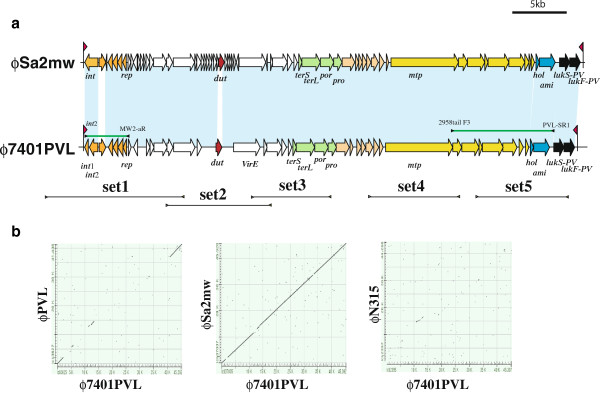
**a. Structural comparisons of the PVL phages.** Structures of phi7401PVL and phiSa2mw are illustrated based on the nucleotide sequences deposited in databases DDBJ/EMBL/GenBank under accession nos. BA000033 for phiSa2mw and AP012341 for phi7401PVL. Red arrowhead indicates the location of *att*P. Black bars indicate the locus of amplified DNA fragments using 5 sets of primers. Green bars indicate the locus of amplified DNA fragment identifying the carriage of gene linkages in phi7401PVL. ORFs are colored as follows: orange, ORFs related to lysogeny; red, a ORF in DNA replication/recombination region with assigned functions; bright green, ORFs related to capsid formation; yellowish orange, ORFs related to head formation; yellowish green, ORFs related to tail formation; blue, ORFs related to cell lysis; black, *lukS-PV* and *lukF-PV*. The locations of the primers are indicated in lines flanked by arrow heads. Nucleotide sequences of the primers are listed in Additional file 2: Table S2. **b**. Comparisons of the two phage genomes with a dot plot analysis. The genome sequence of phi7401PVL was compared to those of phiPVL (group 1 *cos*-site *Siphoviridae*), phiSa2mw (group 2 *cos*-site *Siphoviridae*), and phiN315 (group 3 *cos*-site *Siphoviridae*) using a specialized BLAST at NIBI (http://blast.ncbi.nlm.nih.gov/Blast.cgi). Ordinate indicates the genome phi7401PVL. Abscissa indicates the genomes of three phages, phiPVL, phiSa2mw, and phiN315, in dot plots A, B, and C, respectively.

We determined the nucleotide sequence of a PVL phage lysogenized in a PVL-positive CA-MRSA strain, JCSC7401, isolated in 2006. The strain belonged to ST80 and carried nontypeable SCC*mec* (NT-B). This phi7401PVL was 45,334 bp in length from the rightmost phage attachment site (*att*P-R) to the leftmost site (*att*P-L), in which 44 predicted ORFs larger than 99 bp were identified. The core sequences of 29 nucleotides were located at both ends of phi7401PVL. The G+C content of phi7401PVL was 33.2%, and was comparable to other staphylococcal phages. The overall organization of phi7401PVL was the same as that of previously-reported PVL phages, which consisted of five regions relating to 1) lysogeny, 2) DNA replication/transcriptional regulation, 3) structural modules (the packaging/head and tail), 4) the lysis module, and 5) *lukS*-PV and *lukF*-PV (Figure 1a). The phage was highly homologous to phiSa2mw, which belongs to group 2 of sfi21-like Siphoviridae (Figure 1a and 1b). The entire genome of the phage showed nucleotide identity of more than 95% to that of phiSa2mw. Forty-two of the 44 ORFs were highly homologous to those of phiSa2mw, with the nucleotide identities ranging from 91-100% (Additional file 1: Table S1). The *int* gene was truncated, although it was highly homologous to extant PVL phages. Two ORFs, TUP03 encoding Na/K ATPase and TUP16 encoding dUTPase, were less homologous to phiSa2mw.

In order to determine whether the Tunisian PVL positive strains also carried the same PVL phage as phi7401PVL, we conducted two PCR studies to identify the regions in common with two PVL phages (phi7401PVL and phiSA2mw): a long range PCR study identifying gene linkage *lukS* and the tail gene that can identify PVL phages of the elongated head type and another PCR study identifying the region related to lysogeny (Additional file 1: Table S1 and Figure 1a). In our experiments, all the PVL positive strains were positive in both PCR studies.

## Discussion

### Antibiotic resistance to agents other than β-lactams

The majority of the HA-MRSA isolates were resistant to kanamycin, amikacin and tetracycline. Although the ratio was slightly low (25~55%), these strains were also frequently resistant to tobramycin, gentamicin, erythromycin, quinolones and rifampicin. Recently, it has been reported that rifampicin resistance is related to glycopeptides resistance [25,26]. Since the ratio of rifampicin resistant strains was relatively high, there is a possibility that there might be glycopeptides related to low resistance strains, e.g., hetero-VISA strains. However, glycopeptide resistance is beyond the focus of this study, so we did not examine the details for these findings.

Similar to HA-MRSA isolates, the majority of CA-MRSA isolates were resistant to kanamycin, amikacin and tetracycline, but were susceptible to other antibiotics, except for erythromycin and ciprofloxacin. These data suggest that Tunisian CA-MRSA strains were more resistant to kanamycin, tetracycline and erythromycin than U.S. and Oceanian isolates [27]. In our study, only four CA-MRSA strains were resistant to clindamycin, thus suggesting that clindamycin can be used for the treatment of CA-MRSA infections in Tunisia.

### The PVL phage carried by Tunisian MRSA

The phi7401 carried by a ST80 Tunisian MRSA was highly homologous to phiSa2mw carried by ST1-SCC*mec*IVa MRSA. Only two ORFs, TUP03 and TUP16, showed a lower identity to those of phiSa2mw. Interestingly, TUP03 was identical to ORFs in phi12, phi13, and the bacteriophage in MRSA strains JH1 and JH9, and TUP16 was highly homologous to dUTPase in phiSLT and phi108PVL, with nucleotide identities of 97%. These data suggest that the components of phages were chimeric. Numerous lysogenized phages were induced from the cells of four strains, including JCSC7401 by mitomycin C induction. However, a hybridization experiment with a PVL probe showed that no plaque of the PVL phase was observed. This might have been due to the carriage of a truncated *int*. It seems that lysogenization of the phage occurred early to thus cause a mutation in the phage genome or that the ST80 strains might have an ability to cause a mutation in the *int* to keep the inserted phage genome in the chromosome in a stable form.

### Characteristics of Tunisian MRSA

We determined MLST genotype of all 69 MRSA strains. Our data clearly indicated that all PVL-positive MRSA strains belonged to predicted founder group (FG) 80, which was previously indicated as clonal complex (CC) 80 at the MLST website. In contrast, the PVL-negative MRSA strains belonged to diverse FGs. In this study, we used the FG, which is used at present in the eBurst system on the MLST website. However, by using the old CC system, we can distinguish some lineages more clearly, e.g., ST239 that carries type III SCC*mec* as CC8 and ST5 that carries type II SCC*mec* as CC5, both of which belonged to FG5. Therefore, we listed both the present and former grouping systems in Table 1.

The *agr* types were well correlated with the MLST genotypes; group I, STs 45, 97, 239, 241, 247, and 1819; group II, STs 5 and 22; group III, STs 1, 80, 153, 1440, and new. There was only one exceptional case of a ST80 strain belonging to the *agr* group II. Further experiments including nucleotide sequence determination will be needed to clarify this discrepancy.

The SCC*mec* types of the strains were further determined by multiplex PCR studies, leaving 10 strains still nontypeable. The type IVc SCC*mec* was the most representative one in Tunisia. It was identified both in CA-MRSA (79%) and HA-MRSA (56%). PVL-positive MRSA strains carried SCC*mec* IVc and NT-B, which was supposed to be a novel SCC*mec* type.

The characteristics of Tunisian MRSA strains were also reported by Ben Nejma et al [28]. It has also been reported that the CC80 CA-MRSA strains were predominant clones in Tunisia, similar to many Europeans countries like France, Belgium, and Switzerland [27,29]. The predominance of the type IVc SCC*mec* stain was also reported.

The majority of our CA-MRSA (79%) and HA-MRSA (51%) isolates were *pvl*-positive and belonged to FG80. Our study suggested that the PVL-positive MRSA strains disseminated in Tunisia might be unique to Tunisia or the surrounding countries. Although CC80 PVL positive MRSA strains have been identified in European countries [30], the majority of them carried a type IVa SCC*mec* element or their SCC*mec* subtype was not determined. While two CA-MRSA isolates from Belgium [29] were reported to belonged to ST153-MRSA-IV, the report did not show its subtype.

According to previous studies, PVL-positive MRSA isolates were reported to be associated with an *agr* group III background [27,28,31]. Among our CA-MRSA isolates, the most predominant *agr* group was group III, followed by group II, then group I.

The PVL-positive MRSA clones disseminated in other countries belonged to ST1, ST8, ST22, ST30 and ST59, and carried distinct SCC*mec* elements. Recently, ST30 has been associated with CA-MRSA strains in the United States and in Ireland [27,31] and the ST93 and ST772 strains have been reported in Australia and India, respectively [32,33]. These data suggest that the possibility of simultaneous co-evolution of CA-MRSA organisms in different locations [27] is higher than the possibility of dissemination of a single CA-MRSA clone all over the world. PVL positive strains might therefore have emerged elsewhere and spread in the community and at hospitals.

It is interesting that the PVL-negative MRSA clones were the same MRSA strains isolated in other countries. Two other CA-MRSA isolates belonged to ST5-MRSA-IV which is one of predominant clones in the Netherlands [34]. Concerning the HA-MRSA, the *agr* group I was predominant, as reported previously in Tunisian MRSA [27]. The predominance of a group I background was also reported in United States and in Korea [35,36]. Similar results were obtained in European countries such as Germany and Belgium [36]. Three isolates belonged to the clone ST241-SCC*mec*III. Two belonged to the ST247-SCC*mec*I (Iberian) clone, which is one of predominant clones in Poland [37]. Two other isolates belonged to ST239-SCC*mec*III (Hungarian) clone, which is predominant in Turkey [38].

## Conclusion

Tunisian PVL positive MRSA strains carried the PVL phage, which was highly homologous to phiSa2mw, but distinct in two ORFs. They belonged to FG80 and *agr* group III, and carried type IVc or nontypeable SCC*mec.* Such strains disseminated in the community and might have spread at the Tunisian hospitals by taking over existing MRSA clones, e.g., CC8-SCC*mec*I and CC8-SCC*mec*III.

## Methods

### Bacterial strains

One hundred and fifty-four non-replicated HA-MRSA strains were isolated from 1999 through 2008 at Charles Nicolle Hospital of Tunis. Among them, 41 strains isolated from 2004 through 2008 were chosen based on their resistance profiles. HA-MRSA strains were isolated from mucous pus and blood cultures, puncture fluids, urine, and biomaterials of inpatients.

A total of 28 non-replicated CA-MRSA strains were isolated from January 2004 through June 2008 in two Tunisian hospitals (Charles Nicolle Hospital and Habib Bourguiba Hospital). CA-MRSA strains were isolated from the specimens of the patients with MRSA infections who had not been recently (¬within the past year) hospitalized or undergone a medical procedure (such as dialysis, surgery, catheterization). The CA-MRSA strains were generally recovered from mucous pus, puncture fluids, urine and biomaterials from outpatients. Some MRSA strains isolated from patients within 48 h of hospitalization, e.g., after surgery, in the intensive care unit, in the departments of nephrology, otorhinolaryngology and gynecology, were also included.

### Strain identification

The isolates were identified by the conventional methods (Gram-positive cocci, catalase positive, mannitol fermenting and DNase-positive) and were confirmed to be *S*. *aureus* by their ability to coagulate rabbit plasma (bioMérieux, Marcy l’Etoile, France) and to produce clumping factor (Staphyslide test, bioMérieux). The biotypes were determined using Api20 Staph (bioMérieux, Marcy l’Etoile, France).

### Isolation of MRSA strains

Strains were purified twice, first on MRSA BD select agar (from Baird Dickinson) and then on the Difco Tryptic Soy Agar (TSA) supplemented with an antimicrobial agent (ceftizoxime sodium) from Astellas Pharma Inc, Tokyo, Japan. The detection of the *mecA* gene by PCR was conducted as described previously [39].

### The MIC of oxacilline

The MIC of oxacilline was determined by the agar dilution method according to the Clinical and Laboratory Standards Institute guidelines (CLSI) [40]. *S. aureus* ATCC 29213 was used as a reference strain.

### Antimicrobial susceptibility testing

The antibiotic susceptibility of the isolates was assessed using the disk diffusion method according to the CLSI guidelines, except for pristinamycine, which was used according to the CA-SFM guidelines. The following antimicrobial disks were tested: penicillin G (10UI), oxacillin (1 μg), ampicillin (10 μg), amoxicillin + clavulanic acid (20/10 μg), cephalotin (30 μg), cefoxitin (30 μg), kanamycin (30 μg), gentamicin (10 μg), tobramycin (10 μg), tetracyclines (30 μg), chloramphenicol (30 μg), ofloxacin (5 μg), ciprofloxacin (5 μg), trimethoprim + sulfamethoxazole (1.25/23.75 μg), erythromycin (15 μg), clindamycin (2 μg), vancomycin (10 μg), teicoplanin (30 μg), rifampicin (5 μg), fosfomycin (5 μg) and pristinamycine (15 μg).

### Culture and DNA extraction

The strains were grown on TSB culture at 37°C overnight with shaking. Genomic DNA used as a target for PCR assays was extracted by using a Qiagene kit (QIAamp DNA Mini Kit (250) QUIAGEN. Sciences - US) according to the manufacturer’s instructions.

### SCC*mec* typing

The SCC*mec* elements were typed using two multiplex PCR strategies (M-PCR1 and M-PCR2) which are used for SCC*mec* typing assignment, M-PCR3 was used for the J1 region difference-based subtyping, as described previously [41]. The reference strains used were as follows: NCTC10442(type I), N315(type II), 85/2082(type III), CA05(type IVa), 8/6-3P(type IVb), and 81/108(type IVc).

### Detection of the Panton-valentine leukocidin gene

The carriage of *lukF-*PV and *lukS-*PV genes encoding PVL was examined by PCR as described previously [42].

### *agr* typing

The presence of the accessory gene regulator, *agr,* was determined by multiplex PCR amplifying the hypervariable domain of the *agr* locus, as described previously [43]. PCR amplification was performed in a 50 μl reaction mixture composed of 2U of Ex *Taq* (Takara Shuzo Co., Ltd., Kyoto, Japan), 10 pmol of each primer, 0.2 mM deoxynucleoside triphosphate mixture, 10 ng of chromosomal DNA, 1X reaction buffer (Takara Shuzo Co., Ltd.) and H_2_O. Thermal cycling was set at 30 cycles (30s for denaturation at 95°C, 1 min for annealing at 55°C, and 2 min elongation at 72°C) and was performed with a Gene Amp PCR system 9600 (Perkin-Elmer, Wellesley, Massachusetts).

### MLST

The genotypes were determined by Multilocus Sequence Typing (MLST) according to the procedure used by Enright et al [44]. The alleles of each locus were compared, and sequence types (STs) were assigned based on the *S. aureus* MLST database (http://saureus.mlst.net/).

### *Spa* typing

The typing of the polymorphic region of the protein A gene (*spa*) was performed as described previously [45]. Purified *spa* PCR products were sequenced, and short sequence repeats (SSRs) were assigned using the *spa* database website (http://www.tools.egenomics.com/).

### Determination of nucleotide sequences

Genomic DNA of strain JCSC7401 was extracted with phenol/chloroform and the nucleotide sequences were determined using a 454 genetic analyzer. PCR studies were conducted to amplify the DNA fragment covering the gap of the contigs obtained by the 454 genetic analyzer. The nucleotide sequence of PCR products amplified by long-range PCRs with primer’s pairs listed in Additional file 2 were determined using an ABI sequencer. The nucleotide sequence of phi7401PVL was deposited to the DDBJ/EMBL/GenBank databases under accession no. AP012341.

## Competing interests

The authors declare that they have no competing interests.

## Authors’ contributions

JM, BI, and HA collected strains at Tunisian hospitals. TI, JM, and BI designed the research and prepared the manuscript. KH and HA add the suggestions for the research and preparing the manuscript. JM, MZ, JJ, SL, and HX performed experiments. MZ, JJ and TI contributed for the nucleotide sequencing and data analysis of the PVL phage. All authors read and approved the final manuscript.

## Supplementary Material

Additional file 1 **Table S1.** ORFs in and around phi7401PVLand their similarities to phiSa2mw. Click here for file

Additional file 2 **Table S2.** List of primers used in this experiment. Click here for file
