# Supplementary material for: Molecular characterization of methicillin-resistant Panton-valentine leukocidin positive staphylococcus aureus clones disseminating in Tunisian hospitals and in the community
Source: BMC Microbiol. 2013 Jan 7;13:2. doi: 10.1186/1471-2180-13-2 (PMC3544733; doi:10.1186/1471-2180-13-2)
Supplement: Supplementary file 1 [file 1471-2180-13-2-S2.doc]

Supplementary Table 2. List of primers used in this experiment.

Objectives of primer construction	Primer name	Nucleotide sequence(5'-3')	Size(bp) of PCR product	Location(s) of primer(s)[a)]
**For screening of PVL positive strain**				
lukS,F-PV	PVL-F	ATGTCTGGACATGATCCAA	971	45291−45309
	PVL-R	AACTATCTCTGCCATATGGT		46242−46261
**For long-range PCR to amplify entire region of ϕ7401PVL**				
From chromosome to TUP14 (set1)	7401-chromosomeR	AAGCTACACGTGTATCTGAA		
	CF21	GCACATCATGTCATGCATTT	13500	10673-10692
From TUP12 to TUP19 (set2)	CF18	TAAAGCAATCTGCTGAAGATG		9092-9112
	MW1405R	TGTAGACAGTTCATCAATCAC	9646	18717-18737
From *VirE* to *pro* (set3)	CF34	TGCATGGTTTTCTGACAGTT		16917-16936
	CF49	GGATGCATTTTTAGCATATTG	7280	24176-24196
From TUP30 to TUP34 (set4)	CF56	AGTCTGACATGAGTATTTTC		27650-27669
	2958-M-TR12	CACCTGTTGTATAGTTAACCA	8391	36020-36040
From TUP35 to lukF-PV (set5)	CF77	AAATAGAGCCAAGAGATAGC		37549-37568
	CF92	CTCCACCATAAGAATAACCT	8524	46053-46072
**For PCRs to identify the regions**				
From TUP01 to TUP08	intF2	ATGTTTTCGAGTTTTTGAGTTAG		1606-1628
	MW2-aR	TAAGTTCCTGGTGTCATTCCTAAT	4106	5688-5711
From TUP33 to TUP43	2958tail F3	ATTGATTCAAACTGTTTCTTCTCAGGA		35284-35310
	PVL−SR1	ACGAAGTAGCAATAGGAGTGA	9483	44746-44766

a) Locations of primers were indicated based on the nucleotide sequences of ϕ7401PVL depositted to DDBJ/EMBL/GenBank databases under the accerrion no. AP012341
